# Emergence of Pulmonary Tuberculosis While Recovering From COVID-19 Pneumonia: A Case Report

**DOI:** 10.7759/cureus.44398

**Published:** 2023-08-30

**Authors:** Bhanu Shrestha, Ravi Mahat

**Affiliations:** 1 Department of Respiratory Medicine, Karuna Hospital, Kathmandu, NPL

**Keywords:** tb: tuberculosis, covid pneumonia, hospital-acquired pneumonia, covid 19, copd (chronic obstructive pulmonary disease)

## Abstract

COVID-19 can have different presentations; from asymptomatic to multiorgan involvement. This case report is of an elderly gentleman, with known comorbidities of chronic obstructive airway disease and alcoholic liver disease on treatment. He presented with a history of increasing dyspnea and cough for a few days which was present after cold symptoms, and was admitted for the treatment of severe COVID-19 pneumonia. Later, while he was recovering from COVID-19 pneumonia, his respiratory symptoms worsened. After a thorough evaluation, his sputum smear was positive for acid-fast bacilli and also rifampicin sensitive on GeneXpert assay. With timely diagnosis and appropriate treatment, he recovered from both acute conditions and was sent home on the twentieth day of admission.

## Introduction

COVID-19 commonly presents with flu-like symptoms but it can have a wide spectrum of presentation; from asymptomatic to multiorgan involvement [1]. Direct COVID-19 infection and cytokine storm are widely accepted mechanisms of severe acute respiratory syndrome coronavirus 2 (SARS-CoV-2). However, it can be complicated by superadded infection and hyperoxia-induced acute lung injury [2]. Sometimes drug-induced pulmonary toxicity can mimic COVID pneumonia [3]. Tuberculosis is also a global problem, especially in developing countries, as well as in developed countries due to diabetes and increasing cases of HIV [4]. There are few case reports of coexisting tuberculosis and SARS-Cov-2 since its outbreak [5]. The coexistence of two diseases makes the management of the diseases more challenging.

## Case presentation

A 78-year-old, thin-looking gentleman ex-smoker, a resident of Kathmandu, Nepal, with a known case of chronic obstructive pulmonary disease (COPD) and chronic alcoholic liver disease (CLD), presented to the emergency department (ED) with the complaint of shortness of breath and increasing cough for the last six days. This was preceded by a common cold, cough, and fever 10 days back. The fever had subsided after two to three days. Other family members also had similar symptoms of cold and cough prior to that but they had recovered on their own. Furthermore, there was neither a travel history to other countries nor a history of tuberculosis infection. Similarly, he had taken all of his childhood vaccinations including the bacille Calmette-Guérin (BCG) vaccine, and is a farmer by occupation.

On arrival in ED, his blood oxygen saturation (SpO2) was 80% on room air and was maintained at 3 liters per minute (LPM) and his respiratory rate (RR) was 26 breaths/minute. He was afebrile and his blood pressure was also normal. He also had bipedal edema and inspiratory crackles over subscapular areas on chest examination.

**Figure 1:**
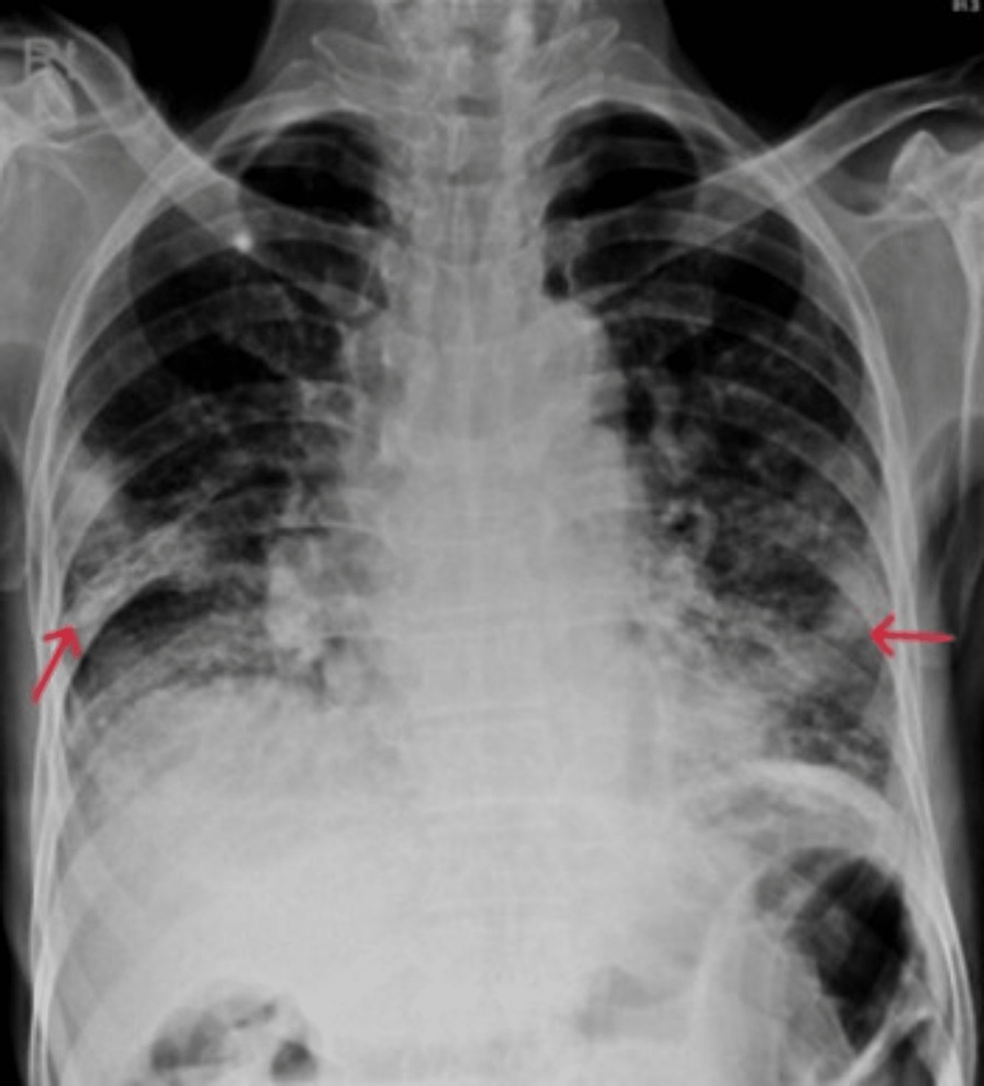
Chest X-ray showing bilateral infiltrates.

On laboratory tests his liver, renal functions, and total leukocyte count (TLC) were normal, hemoglobin (Hb) was 9 g/dl, lymphocytes were 420/mm3, D-dimer was 5.2 mg/L, serum ferritin was 61 ng/ml, and c-reactive protein (CRP) was 28 mg/L. His nasopharyngeal swab RNA test for SARS-CoV2 genes (COVID-PCR) was positive. Initial chest X-ray (CXR) revealed bilateral infiltrates on the mid and lower zones (Figure 1).

**Figure 2:**
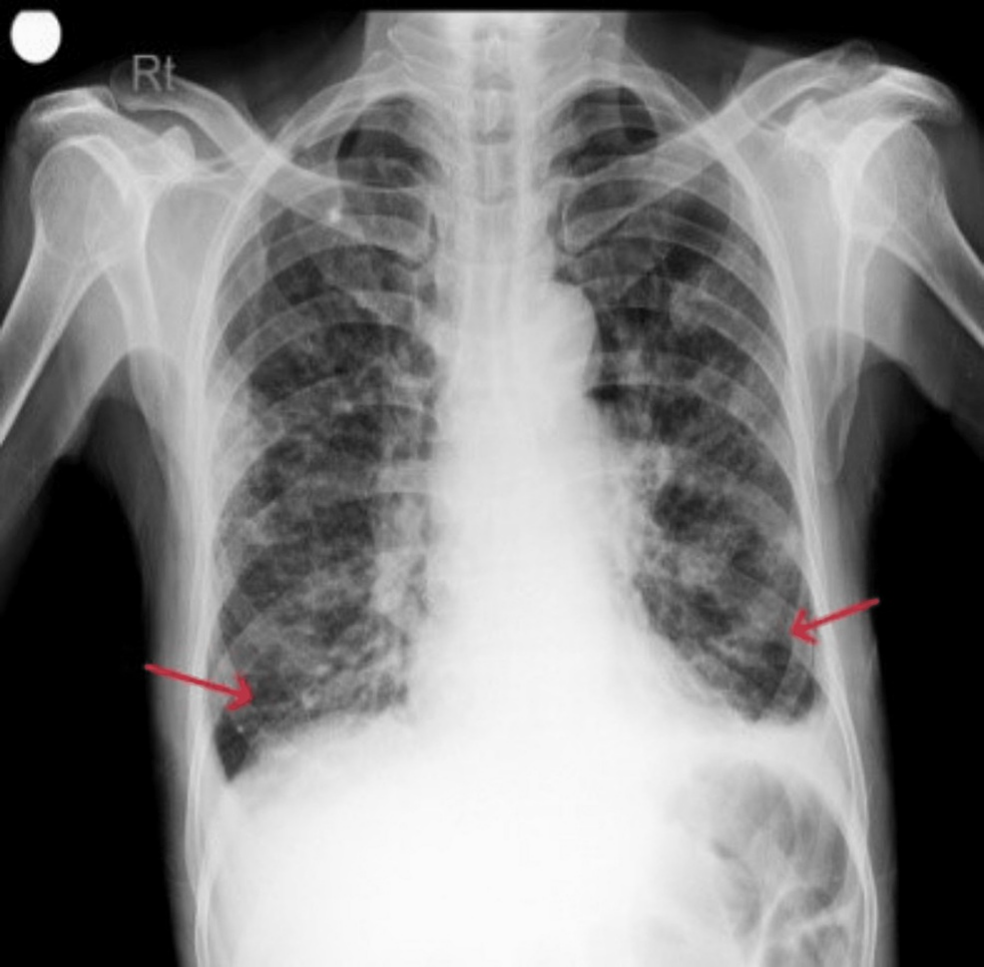
Chest X-ray showing a decrease in the bilateral infiltrates.

Similarly, CXR done on the fifth day of admission showed apparent radiological improvement (Figure 2).

**Figure 3:**
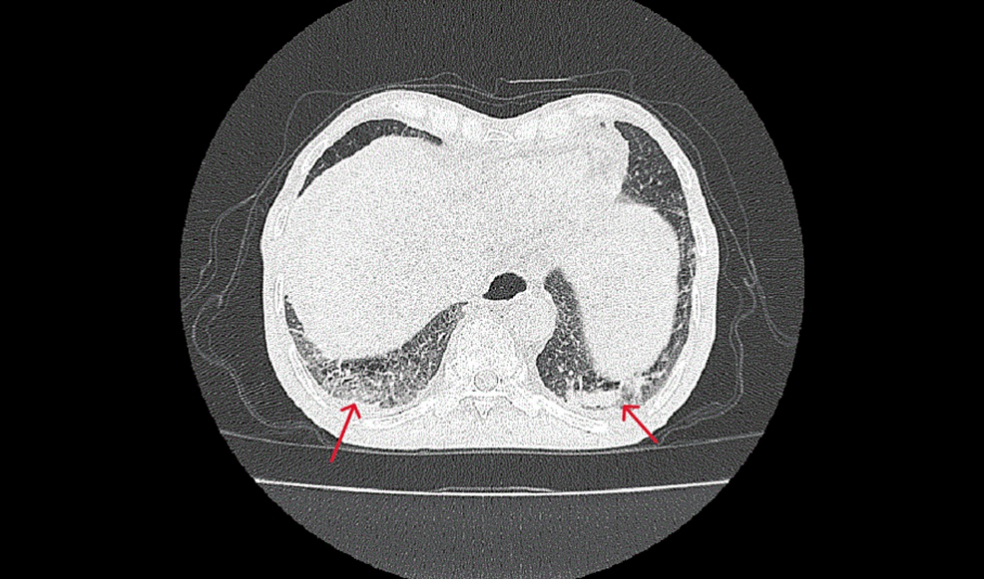
High-resolution computed tomography of the chest revealing bilateral multiple peripheral and some central consolidative lesions on bilateral lungs and emphysematous changes.

High-resolution computed tomography (HRCT) of the chest done on the day of presentation showed multifocal consolidative lesions in bilateral lungs with air-bronchograms, right upper lobe bulla, and moderate ascites (Figure 3).

He was admitted with a diagnosis of COVID-19 pneumonia and managed with dexamethasone, ivermectin, low molecular weight heparin, and other supportive treatments for COPD, CLD, and ascites [6]. There was no growth on blood and sputum culture and sputum Ziehl-Neelsen (ZN) staining was negative for mycobacteria. He improved gradually and after the ninth day of admission repeat COVID-PCR was negative, so he was shifted to the general ward on 1 LPM oxygen supplementation.

**Figure 4:**
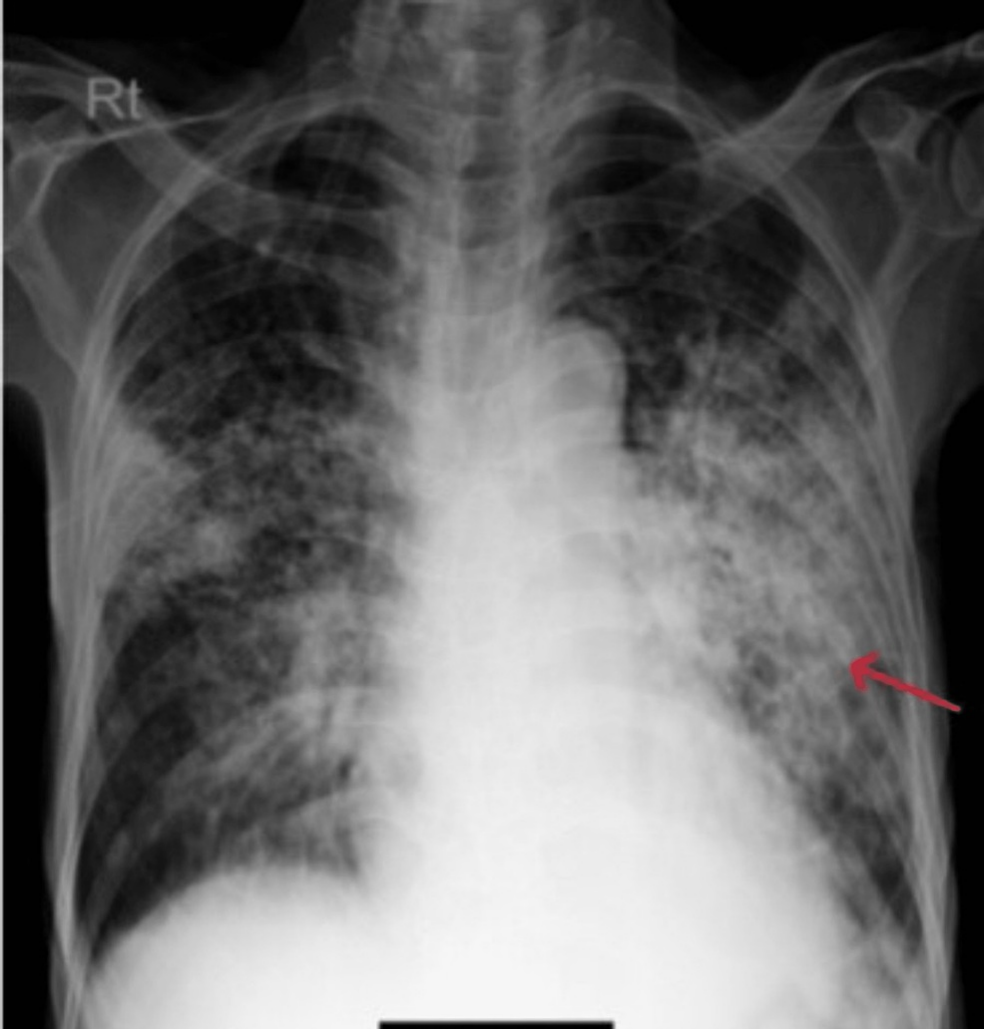
Chest X-ray showing significantly increased infiltrates, more over the left middle zone.

Gradually from the tenth day of admission, his dyspnoea, cough, and sputum production increased. Oxygen requirements had also increased. CXR on the following day showed increased infiltrates on bilateral lung fields, and his SpO2 was 88% at 10 LPM via a non-rebreather mask (Figure 4).

With a provisional diagnosis of hospital-acquired pneumonia (HAP), sputum gram stain and culture, fungal stain, fungus culture, and sputum ZN stain were repeated, and GeneXpert for *Mycobacterium tuberculosis* and resistance to rifampicin (MTB/RIF) assay was also conducted. Broad-spectrum antibiotics were started. He was immediately shifted to the intensive care unit (ICU) and maintained on continuous positive airway pressure (CPAP) and oxygen via a non-breather mask alternatively. His sputum ZN stain showed 3+ acid-fast bacillus (AFB) on consecutive samples; first-line antitubercular therapy (consisting of isoniazid, rifampicin, pyrazinamide, and ethambutol) was started immediately. Later GeneXpert MTB/RIF assay also detected mycobacterial tuberculosis which was rifampicin sensitive as well. He improved gradually with the above treatment and was shifted to the general ward at 2 LPM oxygen via nasal cannula after five days of ICU stay.

## Discussion

SARS-CoV2 is a new strain of coronavirus and many of its effect on our body and immune system is not yet completely known [7]. If a patient is worsening, apart from disease progression, the possibility of superadded infection or reactivation of various infections like tuberculosis or pneumocystis pneumonia should also be considered [8].

Our patient didn't have a history of tuberculosis or contact with a tuberculosis patient in the past and his sputum smear was negative for AFB at the time of presentation. Furthermore, he had taken the BCG vaccine and didn't have any travel history. Later during the course of the hospital stay, due to his worsening clinical condition in spite of earlier improvement, ZN stain was repeated and GeneXpert MTB/RIF was also done along with various infectious workups [9]. The clinical scenario of this particular patient suggests initial COVID-19 infection, which impairs the immune system and may have led to the emergence of tuberculosis [10]. Another possible mechanism of the development of tuberculosis is the use of systemic steroids, which suppress the immune response and may lead to the reactivation of latent infection or a new infection itself [11].

## Conclusions

COVID-19 infection is a multisystem disease. It can take severe form in some infected patients. Superadded infection or reactivation of infectious disease like tuberculosis can also worsen the condition. Thus, clinicians need to maintain a high index of suspicion for pulmonary tuberculosis in such situation, which cannot be explained otherwise.
